# Prenatal arsenic and mercury levels among women practicing geophagy in areas with artisanal and small-scale gold mining activities, Northwestern Tanzania

**DOI:** 10.1186/s12884-023-06174-4

**Published:** 2023-12-12

**Authors:** Jovina Jovine, Elias C. Nyanza, Moses Asori, Deborah SK. Thomas

**Affiliations:** 1 Department of Environmental and Occupational Health, School of Public Health, Catholic University of Health and Allied Sciences, Wurzburg Road, P.O.BOX 1464, Mwanza, Tanzania; 2 Department of Geography & Earth Sciences, University of North Carolina at Charlotte, Charlotte, USA

**Keywords:** Geophagy practices, Pregnancy, Arsenic, Mercury, Artisanal gold mining, Urine, Blood

## Abstract

**Background:**

Artisanal and small-scale gold mining (ASGM) areas potentially pose increased exposure to arsenic and mercury through community contamination, occupations at gold mines, and/or geophagy when soil is locally sourced. This study examined the effects of geophagy, a deliberate soil eating practice, along with community and occupational exposures in ASGM areas on urinary arsenic and blood mercury levels among pregnant women in the Mining and Health Longitudinal Cohort in northwestern Tanzania.

**Methods:**

Data on maternal arsenic and mercury levels were captured for 1056 pregnant women using an unprovoked morning urine samples and dried blood spots respectively. We used a step-wise generalized linear regression model to retain the most relevant covariates for the model. A generalized linear regression model with identity link function was used to predict the effect of geophagy practices on arsenic and mercury levels separately. The model was adjusted using sociodemographic correlates, including maternal age, education level, whether respondents lived in mining or non-mining area, years of residence, marital status, maternal occupation, individual partner’s education, and occupational, and socioeconomic status.

**Results:**

In the adjusted regression model, eating soil during pregnancy increased arsenic concentration by almost 23% (β = 1.229, 95% CI: 1.094, 1.38, *p* < 0.001) and living in mining areas had a 21.2% (β = 1.212; 95% CI: 1.039,1.414, *p* = 0.014) increased risk. Geophagy significantly increased mercury levels by 13.3% (β = 1.133, 95% CI: 1.022, 1.257, *p* = 0.018). Living in areas with ASGM activities was associated with a 142% (β = 2.422, 95% CI: 2.111, 2.776, *p* < 0.0001) increase in blood mercury.

**Conclusion:**

Geophagy practices increased urinary arsenic and blood mercury levels in pregnant women, which was especially true for arsenic when living in areas with ASGM activities. Working in mining = increased risk for blood mercury levels. Community-based environmental health policies should address reductions in occupational and community exposures, along with strategic geophagy reduction interventions.

## Background

Artisanal and small scale gold mining (ASGM) areas potentially pose increased exposure to arsenic and mercury through community contamination, occupations at gold mines, and/or geophagy, deliberately eating soil particularly when soil is locally sourced [1–3]. ASGM activities are commonly conducted with minimal environmental, occupational, or community safety precautions [4], resulting in soil contamination from arsenic, a constituent of gold ore in many areas, and mercury, which is commonly used during gold processing.

Pregnant women who practice geophagy, in contaminated areas may be at increased risk of either acute and/or chronic exposure to arsenic and mercury [2, 5–11]. In a study in Geita District, Tanzania (an ASGM area), geophagy was reported in 46.5% of pregnant women [2]. In the Mining and Health Longitudinal Cohort Study in the same area, results reported high levels of urinary arsenic (38.2 µg/L) and blood mercury (3.8 µg/L) with more than 24% and 76.5% of pregnant women respectively having levels above the human biomonitoring reference values established by the Germany Environmental Survey (GerESIV) of 15 µg/L [9]. However, geophagy was not included as part of the initial analysis.

Geophagy is common in sub-Saharan Africa, with various studies documenting a consumption of soil by pregnant women ranging between 10 and 75% across this region [1, 2, 12, 13]. The deliberate consumption of soil during pregnancy is influenced by traditional, physiological, psychological, medicinal, cultural and religious beliefs [1, 3–5]. Previously, some women reported undertaking geophagy to reduce nausea and morning sickness, to reduce prolonged labor, or to connect the unborn baby with ancestors for protection [1, 2]. In most cases, women reported starting geophagy as a supplement to a meal during the first trimester and early second trimester [1, 2, 5, 13], which is a critical period of gestation with the potential to impair the developing fetus.

While several studies have focused on a variety of health impacts, such as worm infestation, iron defiance anemia, constipation and malabsorption of nutrients [1, 3, 5], fewer have evaluated the exposure to toxic chemical elements from eating soil [2, 5, 14, 15]. Geophagy may introduce toxic chemical elements through ingestion that can endanger the health of the mother and her unborn child. Arsenic and mercury have been linked to adverse reproductive outcomes, neurological disorders, and impaired cognitive development in children [6–8, 16]. In fact, arsenic and mercury are toxic chemicals of major public health concern [8, 16]. Such toxic chemicals cross the placenta and penetrate the blood-brain barrier and evidence suggests that the developing fetus is sensitive to their effects even at low doses [17]. Some studies have indicated that exposure to toxic chemicals, such as arsenic and mercury, may cause adverse pregnancy effects including spontaneous abortion, prematurity, prenatal mortality, low birth weight, decreased fetal head circumference, and congenital malformations [4, 7–9].

While potential environmental exposure has been explored in ASGM areas, to the best of our knowledge, no studies have tested individual arsenic or mercury levels in women while pregnant along with information specific to geophagy practices. To fill the gap, this study examined the effects of geophagy practices and occupational participation in ASGM mining activities on urinary arsenic and blood mercury levels among pregnant women in the Mining and Health Longitudinal Cohort in northwestern Tanzania. The hypothesis was that pregnancy-related geophagy practices would increase mothers’ arsenic and mercury levels, particularly for those residing in communities with ASGM activities, and would be further intensified by working in mining.

## Methods

### Study setting, design and participants

The study settings for the Mining and Health Longitudinal Cohort in northwestern Tanzania have been explained in details elsewhere [9, 18]. Briefly, the Mining and Health Longitudinal Cohort involved two districts: Geita District, an area with intensive ASGM activities, and Magu District, an area without ASGM activities. The settings are similar, with the exception of the gold mining. The two districts are 160 km apart by road (or 150 km by radial distance) and separated by Lake Victoria [9]. The comparison group, women from areas with non-ASGM activity in Magu District, was included to ascertain background levels of exposure in areas with non-ASGM activities.

### Study design and population

This reanalysis is part of the ongoing Mining and Health Longitudinal Cohort study in northwestern Tanzania, which included 1056 pregnant women who were recruited into the study in 2017. The specifics of the study population, including ethical approval are described elsewhere [18]. While the focus of the original study was not geophagy, the survey incorporated a question about soil eating that offered an opportunity to reanalyze the data in the context of existing literature demonstrating deleterious health effects of geophagy from contamination in soil sources.

### Variables measurement

#### Mercury and arsenic levels

For detailed explanation for blood mercury and urinary arsenic levels measurement in pregnant women, see elsewhere [18]. Briefly, total arsenic levels in urine (T-As) among pregnant women was collected using unprovoked morning urine samples during 16–27 weeks gestation age. Gestation age was determined using the last normal menstrual period, as this information was consistently available for all women. Urine samples were analyzed for total arsenic levels using inductively coupled plasma mass spectrometry (ICP-MS) with an ISO 127,025 accredited laboratory method. Polyethylene containers used for collecting urine samples were thoroughly washed with acid to avoid external contamination of the sample. For total mercury blood levels in pregnant women, capillary blood was obtained on a filter paper (Whatman #903) with a finger prick (dried blood spots -DBS). Information on pregnancy and associated behaviors were collected using a modified version of the standardized WHO verbal autopsy algorithm [19], which is available as part of the Mining and Health Longitudinal Cohort Database.

#### Baseline information and geophagy practices among pregnant women

As part of the Mining and Health Longitudinal Cohort, a face-to-face survey was conducted of pregnant women that included a question asking if whether they had eaten soil in the first and second trimester. Because this was not the focus of the initial study, the survey did not include a comprehensive set of questions about geophagy practices, only whether they had practiced or not. In previous studies in this area, women who practiced geophagy typically ate soil consistently, not just one time; so it is reasonable to use this question as a proxy for broader exposure [2]. Data on age of women, occupation, education level, length of residency, and economic level were also collected. Since it is challenging to determine individuals’ wages and/or income per month or per annum, economic status was determined using eight asset items to classify women’s Social Economic Wealth Quintile (SEWQ) based on the Tanzanian context criteria [18, 20]. The eight items included were: (a) whether the family owns the house they live in, (b) whether the roof of the house they are living in is made of iron sheets/concrete or grass, (c) whether they access a protected water source, (d) whether they access electricity and/or solar power, (e) the number of meals the family consume per day, (f) whether the participant is employed or has a business for income, (g) whether the partner is employed or has a business for income) and whether they own any means of transport such as car, motorcycle, or bicycle as described elsewhere [18].

### Statistical analysis

Data were cleaned and analyzed using SPSS IBM statistics version 28 and RStudio version 4.1.3 [21]. Descriptive findings were summarized by mean or median with their measures of dispersions for continuous variables while percentages or proportions were used for summarizing categorical variables. Due to skewed data distribution, the Kruskal-Wallis Test was used to analyze the median differences between groups for mercury and arsenic concentration. Urinary total arsenic (µg/L) and blood total mercury concentration (µg/L) were each included in an analysis as a dependent variable. Due to skewed distribution, total mercury and arsenic concentrations were log transformed to adjust for normality and homogeneity assumption for regression analysis. A forward step-wise linear regression unadjusted analysis was conducted whereby all the variables that were significant in a simple regression (*p*-value < 0.2) were included in the final model using a generalized linear model (GLM) with identity link function to estimate our parameters of associations. Then, the effect of geophagy practices on arsenic and mercury concentration was adjusted for maternal age, education level, whether respondents lived in mining or non-mining area, years of residence, marital status, maternal occupation, individual partner’s education, and occupational, and socioeconomic wealth quantile (SEWQ). Since response variables were log transformed, results from univariate and multivariable linear regression were exponentiated for appropriate interpretation. A *p*-value of < 0.05 was considered statistically significant.

## Results

### Baseline characteristics of pregnant women

Table 1 presents socio-demographic characteristics of the 1056 pregnant women who participated. The mean age was 25.5 (SD = 6.3) years and 32.2% were between 20 and 24 years. Based on marital status, 82% of them were single. For educational status, 71.6% attended primary school and 8.3%secondary school or higher. For occupation, 6.3% were involved in mining activities, 5.8% were businesswomen, 1.7% were public servants, and 85.7% were farmers and casual laborers. Of the pregnant women, 2.7% were residents of the study district for less than two years, 19% had lived in the district for 2–5 years and 78.3% had been the residents for more than 5 years. 18.9% had high social economic wealth quintile (SEWQ), 50.4% were in a moderate category, while 30.7% were in a low SEWQ category. During the study, the mean age at gestation was 25 years (SD = 1.6 years). Of those who lived near the mine, 36% had a history of eating soil during their pregnancy and about 84% had a history of engaging in ASGM activities.

**Table 1:** Socio-demographic status among the study participants

Variables	Geophagy		Non-Geophagy	
*Sociodemographic-Economic Status*	mean	(SD)	mean	SD
BMI of the mother (*kg/m*^*2*^*)*	24.33	3.538	24.42	3.240
The average age of pregnant women (yrs.)	26.40	6.451	24.96	6.169
*The age group of mothers*	*n*	*%*	*n*	*%*
14–18	52	13.6	134	19.9
20–24	115	30.0	223	33.2
25–29	99	25.8	156	23.2
30–34	59	15.4	98	14.6
35–39	46	12.0	45	6.7
40+	12	3.1	16	2.4
*Maternal education*				
No Education/Illiterate	55	14.4	155	23.1
Primary school and above	328	85.6	517	76.9
*Partners’ education*				
No Education/Illiterate	36	9.4	96	14.3
Primary school and above	347	90.6	576	85.7
*Mother has had vocational training.*				
Yes	39	10.2	90	13.4
No	344	89.8	582	86.6
*Years of residence in a respective district*				
< 2	7	1.8	21	3.1
2–5	70	18.3	131	19.5
5+	306	79.9	520	77.4
*Marital status*				
Single	7	1.8	29	4.3
Married/cohabiting	370	96.6	632	94.0
Divorces/separated	6	1.6	11	1.6
*Mother’s occupation*				
Farm/animal keeping	320	83.6	584	86.9
Mining	33	8.6	39	5.8
Business	25	6.5	36	5.4
Public servant	5	1.3	13	1.9
*Partner’s occupation*				
Farm/animal keeping	230	60.1	433	64.4
Mining	109	28.5	134	19.9
Business	37	9.7	85	12.6
Public servant	7	1.8	20	3.0
*Social Economic Wealth Quintile (SEWQ)*				
High	85	22.2	115	17.1
Moderate	221	57.7	311	46.3
Low	77	20.1	246	36.6

### Maternal prenatal total urinary arsenic and total blood mercury concentrations by geophagy and ASGM activities

Tables 2 and 3 summarizes differences in urinary total arsenic and blood total mercury levels among pregnant women in relation to geophagy practice and ASGM activities. The median difference in arsenic levels between mining and non-mining areas was statistically significant (*χ*^*2*^ *=* 12.267; *p* < 0.001). In ASGM areas, there were no significant differences in urinary arsenic levels between women who eat soil during pregnancy and those who did not (*χ*^*2*^ *=* 2. 779; *p* = 0.077). For those who lived in non-ASGM areas, there was a significant difference of arsenic levels based on geophagy practices (*χ*^*2*^ *=* 26.96; *p* < 0.001).

**Table 2:** Maternal total Urinary arsenic levels (T-As) by geophagy practices and ASGM activities (*N* = 1056)

Groups	Median (IQR/%)		
**AGSM Community**			
Practicing geophagy	10.92 (7.12, 17.6)		
Not practicing geophagy	9.90 (5.33, 15.1)		
Working in mining (overall)	10.255 (5.9, 16.06)		
Working in mining (geophagy)	10.35 (6.92, 16.17)		
Working in mining (no geophagy)	9.89 (5.09, 14.85)		
Not working in mining (overall)	11.3 (6.27, 16.17)		
Not working in mining (geophagy)	12.11 (7.12, 17.77)		
Not working in mining (no geophagy)	10.82 (5.97, 15.75)		
***Group comparison***			
Comparison	Median difference	Test-statistic	*p*-value
Geophagy practices vs. non-geophagy	1.02	*χ*^*2*^ = 2.7793	0.077
Mining vs. non-mining area median difference	3.12	*χ*^*2*^ *=* 12.267	< 0.001**
Working in mine vs. not working in mine	1.88	*χ*^*2*^ *=* 4.7066	0.030
**Non-AGSM Community**			
Practicing geophagy	16.05 (10.825, 20.25)		
Not practicing geophagy	5.54 (3.66, 9.45)		
Working in mining (overall)	7.18 (3.81, 14.4)		
Working in mining (no geophagy)	5.72 (3.66, 12.5)		
Working in mining (geophagy)	16.40 (10.83, 19.3)		
Not working in mining (overall)	5.82 (3.3, 14.1)		
Not working in mining (no geophagy)	3.77 (3.02, 5.6)		
Not working in mining (geophagy)	15.2 (11.725, 21.325)		
***Group comparison***			
Geophagy practices vs. non-geophagy	10.51	*χ*^*2*^ *=* 26.964	< 0.0001***
Mining vs. non-mining area median difference	1.36	*χ*^*2*^ *=* 4.267	< 0.0345*

**Table 3:** Maternal total blood mercury levels (T-Hg) by geophagy practices and ASGM activities (*N* = 1056)

Mercury concentration			
***AGSM Community***			
***Groups***	***Median (IQR/%)***		
Practicing geophagy	1.182 (0.83, 1.8)		
Not practicing geophagy	1.195 (0.78, 1.90)		
Working in mine (overall) Working in mine (geophagy)	1.14 (0.79, 1.81) 1.09 (0.81, 1.67)		
Working in mining (no geophagy)	1.2 (0.77, 1.9)		
Not working in mine (Overall)	1.44 (0.9, 2.36)		
Not working in mine (geophagy)	1.765 (1.01, 3.08)		
Not working in mine (no geophagy)	1.21 (0.83, 1.835)		
***Group comparison***			
* Comparison*	*Median difference*	*Test statistic*	*p-value*
Geophagy practices vs. non-geophagy	0.68	*χ*^*2*^ *=* 12.297	< 0.0001
Working in mine vs. not working in mine	0.093	*χ*^*2*^ *=* 4.473	0.1068
***Non-AGSM Community***			
Practicing geophagy	1.28 (0.86, 1.58)		
Not practicing geophagy	0.78 (0.32, 0.9825)		
Working in mine (overall)	0.63 (0.32,1.11)		
Working in mine (geophagy)	1.1 (0.85, 1.45)		
Working in mining (no geophagy)	0.6 (0.312, 0.98)		
Not working in mine (Overall)	0.93 (0.47, 1.51)		
Not working in mine (geophagy	1.49 (1.0725, 1.53)		
Not working in mine (no geophagy)	0.655 (0.43, 0.66)		
***Group comparison***			
Geophagy practices vs. non-geophagy	0.68	12.296	< 0.0001

For blood mercury levels in ASGM areas, there were significant differences in median levels between women who practiced geophagy during pregnancy and those who did not (*χ*^*2*^ *=* 12.297; *p* < 0.0001). There was no median differences in blood mercury levels between those who were involved in ASGM activities and those who were not (*χ*^*2*^ = 4.473; *p* = 0.107). For those who lived in non-ASGM areas, there was significant difference in median blood mercury levels based on geophagy practices (*χ*^*2*^ *=* 12.296; *p* < 0.001).

### Predictors of urinary total arsenic and blood total mercury levels among pregnant women by ASGM and geophagy

Table 4 presents predictors of urinary total arsenic and blood total mercury levels for living and working in ASGM communities and geophagy practices. In the bivariate analysis, results indicate that women who practice geophagy during pregnancy had increased arsenic levels by 20.2% (β = 1.202; 95% CI: 1.078, 1.341, *p* < 0.001). Those who lived in ASGM areas had increased urinary arsenic levels of 31.3% (β = 1.313; 95% CI: 1.118, 542, *p* < 0.001). Having not gone to school increased arsenic levels by nearly 29% (β = 1.285, 95% CI: 1.125, 1.467; *p* < 0.001). Furthermore, urinary arsenic levels for women working in mining had an increased risk of 16% (β = 1.159; 95% CI: 0.939, 1.431, *p* = 0.169) even though association was not statistically significant. Increased socioeconomic wealth quantile was associated with a 3.3% reduction in urinary arsenic levels (β = 0.967, 95% CI: 0.945, 0.99; *p* = 0.004). However, maternal age (β = 1.004; 95% CI: 0.996, 1.013, *p* = 0.283) was not statistically associated with urinary arsenic levels.

**Table 4:** Association between geophagy practices among pregnant women, and arsenic and mercury levels (*n* = 1056)

Total Urinary Arsenic concentration (µg/L)						
Explanatory Variables	Unadjusted			Adjusted		
	exp (coefficient^β^)	(95% CI)	*p-value*	exp (coefficient^β^)	(95% CI)	*p-value*
Age group of mothers	1.004	(0.996, 1.013)	0.283	1.003	(0.995, 1.012)	0.455
Mother education						
At least primary education	1			1		
No formal education	1.285	(1.125,1.467)	< 0.001***	1.278	(1.118,1.461)	< 0.0001***
Total SEWQ	0.967	(0.945, 0.99)	0.004**	0.968	(0.946,0.991)	0.006**
History of eating soil during pregnancy						
No	1			1		
Yes	1.202	(1.078,1.341)	< 0.001***	1.229	(1.094,1.38)	< 0.0001***
Living in ASGM area						
No	1			1		
Yes	1.313	(1.118, 1.542)	< 0.001***	1.212	(1.039,1.414)	0.014*
Working in mining						
No	1			1		
Yes	1.159	(0.939, 1.431)	0.169	1.09	(0.877,1.355)	0.438
Total Blood Mercury concentration (µg/L)						
Age group of mothers	0.993	(0.985,1.001)	0.076^a^	0.993	(0.986, 1.012)	0.066^a^
Mother education						
At least primary education	1			1		
No formal education	1.161	(1.021,1.321)	0.023*	1.126	(0.999, 1.271)	0.05*
Total SEWQ	0.968	(0.948,0.988)	0.002**	0.978	(0.958, 0.998)	0.032*
History of eating soil during pregnancy						
No	1			1		
Yes	1.233	(1.109, 1.371)	< 0.001***	1.133	(1.022, 1.257)	0.018*
Living in ASGM area						
No	1			1		
Yes	2.412	(2.112, 2.728)	< 0.0001***	2.422	(2.111, 2.776)	< 0.0001***
Working in mining						
No	1			1		
Yes	1.518	(1.107, 2.083)	0.01*	1.307	(1.074, 1.59)	0.007**

Beta is exponentiated

*p < 0.05, **p < 0.01, ***p < 0.00

^a^for marginally significant

In the adjusted model, geophagy practices during pregnancy increased arsenic levels by almost 23% (β = 1.229, 95% CI: 1.094, 1.38, *p* < 0.0001). Increased socioeconomic wealth quantile reduced arsenic levels by 3.2% (β = 0.968, 95% CI: 0.946, 0.991, *p* = 0.006). Having not gone to school and living in areas with ASGM activities were associated with a 28% (β = 1.278; 95% CI: 1.118, 1.461, *p* < 0.001) and 21.2% (β = 1.212; 95% CI: 1.039, 1.414, *p* = 0.014) increase in urinary arsenic levels respectively. However, working in the ASGM (β = 1.09, 95% CI: 0.877, 1.355, *p* = 0.438) and maternal age (β = 1.003, 95% CI: 0.995, 1.012, *p* = 0.455) were not statistically associated with increased urinary arsenic levels.

For maternal blood mercury levels in the unadjusted model, women who practiced geophagy had increased levels of 23.3% (β = 1.233; 95% CI: 1.109, 1.371, *p* < 0.001). There was a significant 141% increase in blood mercury levels among pregnant women who lived in areas with ASGM activities (β = 2.412; 95% CI: 2.112, 2.728, *p* < 0.0001). Individual pregnant women who had not gone to school were associated with a 16% (β = 1.021,1.321, *p* = 0.023) increase in blood mercury levels. Having a higher socioeconomic wealth quantile was a protective factor for blood mercury level among pregnant women (β = 0.968, 95% CI: 0.948, 0.988, *p* = 0.002). Pregnant women who worked in ASGM had an increased blood mercury concentration of 51.8% (β = 1.518, 95% CI: 1.107, 2.083, *p* = 0.01). Maternal age did not statistically predict blood mercury levels (β = 0.993, 95% CI: 0.985, 1.001, *p* = 0.076). In the adjusted model, geophagy practices significantly increased blood mercury levels by 13.3% (β = 1.133, 95% CI: 1.022, 1.257, *p* = 0.018). A high socioeconomic wealth quantile was associated with 2.2% reduction in blood mercury level (β = 0.97, 95% CI: 0.958, 0.998, *p* = 0.032). Having not gone to school increased blood mercury levels by 12.6% (β = 1.126, 95% CI: 0.999, 1.271, *p* = 0.05). Living in areas with ASGM activities and working in the mines were associated with a 142% (β = 2.422, 95% CI: 2.111, 2.776), *p* < 0.0001) and a 31% (β = 1.307, 95% CI: 1.074, 1.59, *p* = 0.007) increase in mercury levels respectively.

## Discussion

Exposure to toxic chemicals through geophagy practices is an alarming and salient public health issue. Several studies have documented the potential risks of geophagy practices in different parts of the world through the analysis of soil content eaten by pregnant women [2, 14, 15, 22–25]. This study provides a contribution in the ongoing discourse on geophagy and environmental exposure by examining the arsenic and mercury levels in pregnant women.

The substantial percentage (36%) of pregnant women practicing geophagy in the current study is fairly similar to previous studies in Tanzania in the same geographic area that reported around 46% [2]. The 10% decrease may be attributed to the knowledge translation and health promotion campaigns that are ongoing via different media and antenatal care clinic sessions that discourage the practice of soil eating especially during pregnancy or perhaps because of differences in study populations or regions. A study conducted in Ghana where 21.2% of the pregnant women ate white clay as their nutritious food [26] and in South Africa where 22.5% of the pregnant women practiced geophagy [27] illustrate the extent of the practice across sub-Saharan Africa. Social cultural norms and taboos, such as reducing prolonged labor, have been reported as drivers for geophagy practices [2].

Community contamination may contribute significantly to the increased arsenic and mercury levels [28], particularly if women eat soil that they take from the local ground. Pregnant women in ASGM communities who are not directly involved in mining may still be exposed to arsenic and mercury through the soil they eat, water they drink, the food they eat, and the air they breathe [29, 30]. In some ASGM operations, the gold mineral matrix is mostly contained in arsenopyrite (FeAsS), orpiment (As2S3), and realgar (As4S4) where liberation of gold through mechanical and chemical means results in the release of arsenic into the environment [30]. Mercury is used in the amalgamation process to bind gold [29, 30]. For instance, in 2005 up to 243µgAs/L was detected in water sediments in one of the drainage areas in Geita [31]. A follow up study in 2014 reported total arsenic and mercury in soil sediments to range between 183 and 20,298 and 5.8–1751 µg/kg, respectively [30]. Environmental contamination from ASGM activities creates a broader community risk of exposure, compounded by geophagy practices when soil is locally sourced. Simply living in an ASGM community increased the risk of arsenic and mercury blood levels significantly in this current study. This is consistent with studies in Ghana, Iran, and Tanzania where the ASGM tailings were reported to have arsenic and mercury levels above the safety levels for soil [28–30].

Increased regulations, educational campaigns, informed choice among consumers, reviewing marketing strategies, and continued research would reduce exposure to mothers and their unborn babies. We also recommend that if soil materials are sold that are used for geophagy, they should be accompanied by special packaging with appropriate health key messages as per the Tanzania Food, Drugs and Cosmetics Act 2015 requirements [32, 33]. Sales of soft stone/sticks (popularly known as *Pemba*) as geophagic materials should be regulated to protect consumer health. Producers should be empowered, and where possible assisted by the respective government authorities, to test the toxicity and/chemical contents of edible soil so as consumers are well informed on their choices. The Tanzania Food Labelling Regulations 2006 [34] and the Tanzania Food, Drugs and Cosmetics Act 2015 [32, 33] both require that all pre-prepared packaged foods and drinks manufactured, processed, pre-packed or packed in Tanzania must clearly display labels and content for consumed products on the pack.

### Limitation of the study

The study obtained information from pregnant women who managed to reach to the health facility for antenatal care clinics (ANC) service. Thus, some pregnant women may not have attended ANC during the cohort participants recruitment. This could have resulted in an underestimation of the magnitude of exposure levels as some women with higher levels may have not attended the antenatal care clinics. In addition, geophagy practices among pregnant women were self-reported and so may over- or under- report the practices. Because geophagy is common practice in Tanzania the tendency to underreport might have been mitigated because women are not ashamed to talk about it [2, 5]. Further, the survey did not capture the amount or frequency of soil eaten by the pregnant women, though pregnant women have reported eating soil at least three times a day in this area with the soil ingestion rate (amount of soil eaten on average by women per day) estimated at 62.5 g/day, occurring mostly during the first and second trimester of their pregnancy [2]. The study did not consider other medical conditions or nutrition status that could affect the pregnant women maternal prenatal urinary arsenic and blood mercury levels. x.

## Conclusion

Geophagy during pregnancy is a public health concern regardless of economic status and level of education because of increased exposures toxic chemicals, such as arsenic and mercury. Pregnancy-related geophagy elevated concentration of urinary arsenic and blood mercury levels in this study. Further, geophagy intensified the risk when living in ASGM area and working in mining, suggesting a compounding effect from occupational, community, and geophagy-associated exposures. Interventions to reduce maternal exposure to toxic heavy metals should consider behavioral, environmental, and occupational factors jointly, including addressing geophagy during pregnancy through reduction campaigns and improved monitoring of soil sold in markets.

## Data Availability

All data and materials used are available upon request to the Corresponding author via e-mail: elcnyanza@gmail.com; Department of Environmental and Occupational Health, School of Public Health, Catholic University of Health and Allied Sciences, Wurzburg Road, P.O.BOX 1464, Mwanza, Tanzania.
